# Digital Detox Strategies and Mental Health: A Comprehensive Scoping Review of Why, Where, and How

**DOI:** 10.7759/cureus.78250

**Published:** 2025-01-30

**Authors:** Sajita Setia, Frank Gilbert, Michelle L Tichy, Julia Redpath, Neha Shahzad, Marisa E Marraccini

**Affiliations:** 1 Department of Health and Medical Education, Transform Medical Communications Limited, Auckland, NZL; 2 Department of Social-Emotional Learning, Transforming Life LLC, Wilmington, USA; 3 Department of Psychology, Norfolk State University, Norfolk, USA; 4 Department of Psychology, Alfred University, New York, USA; 5 School of Education, University of North Carolina Chapel Hill, Chapel Hill, USA

**Keywords:** digital dementia, digital detox, digital downtime, digital technology, mental health, phubbing, problematic smartphone use, problematic social media use, scoping review, social media

## Abstract

Excessive use of social media (SM) platforms and digital technology (DT), often driven by habitual scrolling due to adaptive feed experiences, has been linked to anxiety, sleep disturbances, and obsessive-compulsive behaviors while also exacerbating mental health concerns. Yet, the role of "digital detox", defined as a voluntary reduction or temporary cessation of device use, remains only partially understood as both a clinical and lifestyle intervention. This comprehensive scoping review was conducted to consolidate existing research on digital detox interventions and evaluate contextual factors that may influence their effectiveness for mental health and well-being.

A targeted keyword search for "digital detox" was conducted in the PubMed database on December 12, 2024, yielding 34 initial results. This review followed the approach recommended by the Preferred Reporting Items for Systematic Reviews and Meta-Analyses Extension for Scoping Reviews (PRISMA-ScR) to identify, screen, and extract evidence from relevant studies as per pre-specified inclusion criteria. A total of 14 studies were found eligible, and data from these studies and their relevant references (totaling 640 citations) were extracted and synthesized. Our findings suggest that digital detox interventions may alleviate depression and problematic internet use, and individuals with higher baseline symptom severity appear to derive higher benefits. However, the impact on broader outcomes such as life satisfaction and overall well-being remains variable. Divergent intervention approaches, ranging from short-term SM abstinence to sustained, moderate device restrictions and individual differences in baseline severity of symptoms, coping styles, environmental pressures, and support systems, may contribute to different outcomes across various studies and systematic reviews. Overall, age, gender, baseline mental health, and range and duration of DT usage prior to detox are the key variables that may determine the effectiveness of digital detox interventions. Tailored DT usage in moderation, aligned with each individual's age, developmental stage, and academic needs, has greater benefits among younger populations, particularly adolescents and young adults, while mindful and regulated SM use is especially advantageous for female populations. However, other populations could also benefit, provided interventions address self-regulation challenges specific to adult lifestyles.

Given the growing global prevalence of problematic smartphone use (PSU) and its documented comorbidity with psychiatric disorders, digital detox strategies have the potential to be integrated into clinical recommendations and policy initiatives. However, a framework for assessing intervention quality and long-term outcomes is essential.

## Introduction and background

In today's digital era, social media (SM) platforms and digital technology (DT) have become an integral part of our lives; these serve as primary avenues for communication, self-expression, academics, and work [1-3]. However, these platforms are often engineered to exploit human desires through features such as adaptive feed experiences, which refer to personalized, algorithm-driven content [3]. While this can create a highly engaging, customized experience, it also encourages habitual scrolling and unregulated prolonged device use by continuously presenting content that the user finds appealing or challenging to ignore [4].

Problematic smartphone use (PSU), defined as excessive or uncontrolled smartphone behavior that may lead to harm or impair daily functioning, often affects younger populations [5]. A systematic review and meta-analysis of 109 studies from 2012 to 2022 covering a total of 97,748 individuals estimated the global pooled prevalence of PSU at 37.1% (95% confidence interval (CI): 33.5%-40.8%), showing a steady rise over time despite regional and measurement variations [6]. PSU and unregulated SM use are linked with a range of adverse outcomes, including body image concerns and diminished self-esteem, mental health challenges, obsessive-compulsive behaviors, poor sleep quality, reduced school performance, and lower productivity [7-11]. Extensive research supports this concerning trend, with meta-analyses showing significant correlations between problematic SM use and increased levels of depression, anxiety, and stress [12,13], as well as a link between PSU and a decline in academic achievement [14].

A key factor contributing to these problems is technostress, which arises from the excessive or misuse of technology [15]. Technostress manifests as negative emotional and physiological responses, including anxiety, irritability, frustration, and exhaustion, and is driven in large part by the "fear of missing out" (FOMO). FOMO compels individuals to remain continuously connected with SM or DT in order to avoid missing important information or social interactions [15]. This drive is further reinforced by immediate gratification through the rewarding effect of browsing, e.g., likes and comments and continuous streams of novel content on SM, which stimulates the dopaminergic system, reinforcing prolonged engagement and addictive behaviors [16,17].

DT serves as a primary means of communication for young people, who heavily rely on images to express themselves. The rise of DT and SM has intensified the appearance culture, promoting idealized models and fostering unregulated behavior and excessive social and body image comparison [18,19]. This digital environment exploits the vulnerabilities of adolescents, stimulating desires for experimentation and reinforcing cultural beliefs that can lead to deviant or pathological behaviors related to self-esteem and mental health [18]. For instance, a secondary analysis of the Adolescent Brain Cognitive Development (ABCD) Study found that mobile phone ownership and frequent SM use among pre-teens were associated with increased risks of substance use, including alcohol, nicotine/tobacco, and cannabis, over an 18-month period [20]. Additionally, excessive smartphone use is associated with poor sleep quality and bedtime and academic procrastination [21]. Research involving school and university students from several countries has revealed that excessive SM and DT use is significantly associated with poor sleep quality, intermittent/anxious sleep patterns, and daily function [19,21-24].

Despite these concerns, SM remains an integral part of our daily lives, offering benefits such as enhanced social connections, reduced loneliness, and safe spaces for marginalized groups such as LGBTQ+ individuals [25]. In response to rising mental health issues linked to digital use [26,27], many experts advocate for bans and severe restrictions on SM and DT [28,29]. In Australia, children under 16 will be prohibited from using various SM platforms by 2025 following the federal parliament's legislation through the Online Safety Amendment (SM Minimum Age) Bill 2024, which aims to enhance protections during critical developmental stages [30]. However, a recent study from South Australia has indicated that such restrictions may have limited short-term benefits and do not address the underlying psychological mechanisms driving problematic phone use [31]. Instead of merely enforcing bans, experts suggest leveraging targeted emotion regulation strategies to help adolescents navigate the digital social environment effectively [25,31-34]. Additionally, escalating mental distress is not limited to those under 16; a meta-analysis assessing the global prevalence of depression and anxiety symptoms among college students found high rates, with 33.6% experiencing depression and 39% reporting anxiety symptoms [35]. These findings necessitate the need for intervention programs that focus on enhancing self-regulation skills to reduce smartphone addiction and improve sleep quality and overall physical and mental well-being among all youth populations, including adolescents and young adults [36]. Addressing these challenges through targeted personalized digital detox strategies and emotional regulation, rather than exclusive restrictive measures, may offer a more practical approach to mitigating the mental health impacts of DT use [36].

The concept of digital detox, which refers to disconnecting DT and SM use, has recently emerged as a possible way to address these issues [15,37]. However, its definition varies among experts and citations, and usually, terms such as break, abstinence, disconnection, timeout, detox, or unplugging are used [38]. The clinical application of digital detox, which implies temporarily and voluntarily reducing or eliminating DT and SM use, has recently been proposed, but mainly for the working population [15]. Although initial findings and anecdotal reports suggest that digital detox practices may improve mental well-being, the evidence is still fragmented. Many concepts are not fully understood, such as preferred strategies, support systems, and the populations that could benefit the most [15,39]. As of December 2024, there was no Medical Subject Heading (MeSH) on PubMed for digital detox [40]. This comprehensive scoping review seeks to consolidate and clarify what is currently known, identify key gaps in the research, and guide the development of future systematic reviews, practical takeaways, and strategies to promote mental health in our increasingly connected world.

The key objectives of this review are to (i) examine the range of mental health and well-being indicators influenced by digital detox and (ii) determine the contextual factors that may modulate the priority or impact of digital detox strategies and interventions. We hypothesize that digital detox interventions employ a variety of methodological approaches and outcome measures to evaluate their effects on mental health and well-being. Additionally, we anticipate that specific characteristics of these interventions, such as type, duration, modality, and support mechanisms, along with contextual factors such as demographic characteristics, baseline technology use, baseline well-being, and cultural context, influence their effectiveness.

A previous systematic review found that only about 30% of the eligible studies related to digital detox keywords across multiple databases were of high quality [38]. Given the emerging nature of the digital detox concept and the absence of a dedicated MeSH term for "digital detox" in PubMed as of December 2024, we utilized a focused keyword search using all fields for the keyword "digital detox" within the PubMed database to capture a comprehensive list of relevant and high-quality peer-reviewed medical literature.

## Review

A literature search conducted using the keyword "digital detox" on the PubMed database without any filters on December 12, 2024, yielded 34 results. Table 1 details the eligibility criteria for screening these 34 citations for data extraction.

**Table 1:** Eligibility criteria for data extraction The search included articles on PubMed published from inception until December 12, 2024.

Inclusion criteria	Exclusion criteria
Publication type	
Original research (quantitative, qualitative, mixed-methods) and review articles (systematic, scoping, or narrative)	Commentary, opinion pieces, editorials, letters, news, and conference abstracts
Focus	
Studies or reviews that include any characteristics and implementation components of digital detox strategies to improve mental health or well-being	Studies or reviews focusing solely on individual digital detox apps or only including context related to forced digital overuse (e.g., due to lockdowns or isolation during the pandemic)
Outcomes	
Studies or reviews, including at least one measure assessing the impact of digital detox on mental health or well-being (e.g., anxiety, depression, stress, digital dementia, cognitive burden, life satisfaction, and subjective well-being)	Studies or reviews that do not include the impact of digital detox on any mental health or well-being outcomes (e.g., focusing solely on reduction in screen time, productivity, educational performance, or physical health)

This review followed a structured approach to identify, screen, and extract evidence from relevant studies in accordance with the Preferred Reporting Items for Systematic Reviews and Meta-Analyses Extension for Scoping Reviews (PRISMA-ScR) [41]. Two independent reviewers conducted the initial Phase 1 (title/abstract) and subsequent Phase 2 (full-text) screening. Two reviewers then completed data extraction, and two additional reviewers performed quality checks to ensure accuracy and consistency. Any discrepancies were resolved through discussion or consulting a third reviewer when necessary.

A total of 16 citations were excluded from Phase 1 screening (review of abstract) due to their lack of focus related to the aims or objectives of this scoping review. Specifically, 10 citations did not include any characteristics and implementation components of digital detox strategies to enhance well-being or mental health [42-51]. Additionally, one study was excluded as it focused solely on an individual digital detox app [52]. Some articles addressed forced digital overuse, such as that experienced during lockdowns or isolation periods during the pandemic [53-55], and a few of them also included outcomes unrelated to well-being or mental health [56,57]. Two review articles were excluded from Phase 2 screening as the non-inclusion of any characteristics and implementation components of digital detox strategies to enhance well-being or mental health was only reflected in full-text review [58,59]. Two additional studies were excluded from Phase 2 screening of full texts as the digital detox interventions were studied only for screen time reduction and not for effect on mental health or well-being measures [60,61].

**Figure 1:**
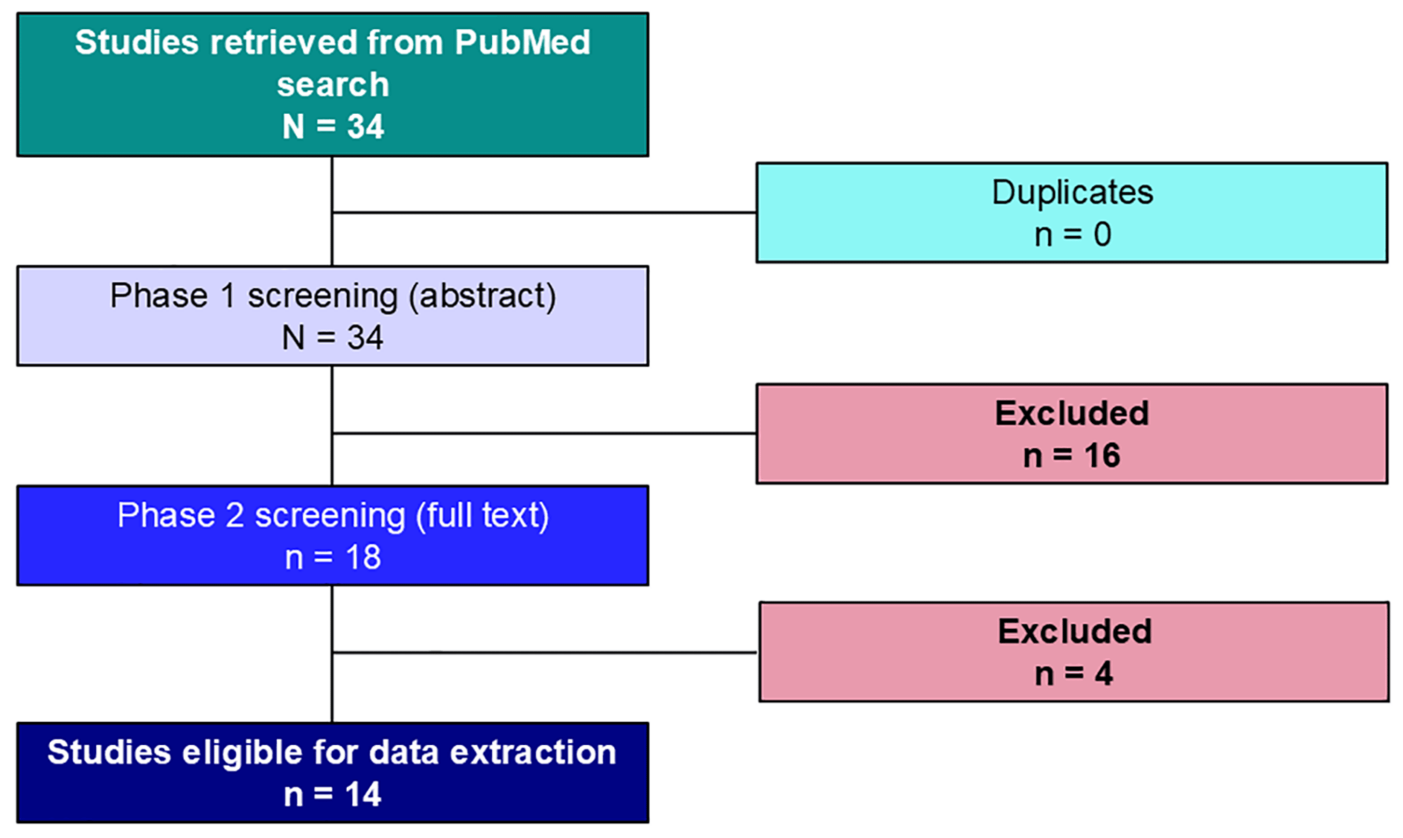
PRISMA flowchart PRISMA: Preferred Reporting Items for Systematic Reviews and Meta-Analyses Image credit: Dr. Sajita Setia (corresponding author)

A total of 14 citations met the eligibility criteria and were deemed eligible by two independent reviewers [62-75]. Two additional reviewers subsequently extracted data to formulate the narrative for this review, with quality checks conducted by a third reviewer. One of the included original quantitative studies, "Digital detox: the effect of smartphone abstinence on mood, anxiety, and craving", which was published in 2019 [74], issued a corrigendum in 2020 [75]. Data were extracted from the original articles and verified with the corrigendum to ensure accuracy and completeness. Figure 1 illustrates the PRISMA flowchart for the overall results of both Phase 1 and Phase 2 screenings.

The narrative of this scoping review (along with practical interpretations and strategies for implementing digital detox to enhance well-being and mental health) was developed using the key findings and interpretations of all 14 eligible studies [62-75] and their associated references, yielding a total of 640 citations considered for this review.

Table 2 provides a concise overview of each included study, outlining their article types, study objectives, methodologies, and relevance to the current scoping review.

**Table 2:** Summary of eligible citations in this review DT: digital technology, SM: social media, PRISMA: Preferred Reporting Items for Systematic Reviews and Meta-Analyses

Study title/publication	Article type	Study objectives and methodology	Relevance in medicine and research
Understanding digital dementia and cognitive impact in the current era of the internet: a review [62]	Narrative review	Reviewed 78 high-quality studies to provide a detailed overview of the research on digital dementia and cognitive impacts due to DT	Highlights the need for digital detox strategies to mitigate cognitive decline associated with DT use, emphasizing the importance of reducing screen time and enhancing cognitive awareness
Partner phubbing and sleep quality: serial mediation models with relationship satisfaction and perceived stress [63]	Original research (quantitative)	Examined how relationship satisfaction and stress mediate the impact of partner phubbing on sleep quality among adults in long-term relationships	Supports the clinical importance of digital detox in improving relationship dynamics and sleep quality, underscoring the necessity for interventions that address interpersonal digital behavior
Digital detox and well-being [64]	Narrative review	Summarized the latest findings on the impact of digital detox on well-being, drawing from a broad range of recent studies	Demonstrates the effectiveness of digital detox interventions in enhancing well-being, calling for more precise measures and standardized tools in future research
Impacts of digital social media detox for mental health: a systematic review and meta-analysis [65]	Systematic review	Synthesized findings from several studies precisely using PRISMA guidelines to assess the impacts of digital detox on mental health variables such as stress and life satisfaction	Indicates the mixed effects of digital detox on various mental health outcomes and suggests the necessity for long-term studies to define effective strategies
Twitter discussions on #digitaldementia: content and sentiment analysis [66]	Original research (quantitative)	Analyzed public perceptions and concerns about digital dementia through a contextual analysis of Twitter discussions	Provides insight into public concerns about digital overuse and its cognitive effects, highlighting the societal call for effective digital detox measures
A literature review on holistic well-being and dopamine fasting: an integrated approach [67]	Narrative review	Reviewed literature on the broad impacts of digital overuse on holistic well-being to provide an overarching synthesis of the field	Calls for a balanced digital lifestyle and the incorporation of detox strategies to mitigate the adverse impacts of digital overuse on overall health
A comprehensive review on digital detox: a newer health and wellness trend in the current era [68]	Systematic review	Explored the breadth of digital detox research, focusing on its cognitive and emotional benefits through an extensive review process	Discusses the varied applications of digital detox and its potential benefits, stressing the need for integrated approaches in mental health interventions
Taking a break: the effects of partaking in a two-week social media digital detox on problematic smartphone and social media use, and other health-related outcomes among young adults [69]	Original research (mixed-methods)	Investigated the immediate psychological impacts of engaging in a digital detox through a mixed-methods approach	Highlights the immediate benefits of digital detox for mental health and suggests the need for further research on its long-term impacts
Restricting social networking site use for one week produces varied effects on mood but does not increase explicit or implicit desires to use SNSs: Findings from an ecological momentary assessment study [70]	Original research (quantitative)	Assessed the effects of reducing social networking site use on well-being over a one-week period	Demonstrates that reductions in SM use can lead to improvements in well-being, with the potential for long-term benefits
Digital wellbeing: the need of the hour in today's digitalized and technology driven world [71]	Narrative review	Examined the negative impact of excessive digital engagement on various aspects of personal health	Stresses the importance of digital well-being initiatives and comprehensive public health strategies to address digital overuse
Characteristics of social media 'detoxification' in university students [72]	Original research (quantitative)	Explored characteristics of SM detoxification behaviors among university students	Findings suggest that intentional SM detox could potentially improve mental well-being and functionality
Does digital detox work? Exploring the role of digital detox applications for problematic smartphone use and well-being of young adults using multigroup analysis [73]	Original research (quantitative)	Investigated the psychological impacts of taking breaks from digital devices and social networking platforms	Highlights the potential benefits of digital breaks, suggesting that their effectiveness depends on individual and contextual factors
Corrigendum and main article "Digital detox: the effect of smartphone abstinence on mood, anxiety, and craving" [74,75]	Original research (quantitative)	Analyzed data to explore the relationship between smartphone use and cognitive health with an erratum correcting previous findings	Reinforces concerns over smartphone-induced cognitive impairments and the potential cognitive benefits of digital detox interventions

Range of mental health and well-being indicators influenced by digital detox interventions: why digital detox matters?

A growing body of evidence suggests that digital detox interventions, i.e., voluntary breaks or reduced and mindful usage of digital devices, can positively influence various mental health and well-being indicators, including stress, anxiety, depression, subjective well-being, and life satisfaction [62,64,65,71]. One emerging concept is "digital dementia", wherein excessive reliance on digital devices (e.g., smartphones) may also contribute to attention deficits, memory loss, and cognitive overload, potentially impairing sleep quality, emotional regulation, and performance at school or work [62,66]. By deliberately reducing digital use, along with applying mindful approaches to digital usage, individuals can aim to restore a healthier "tech-life" balance, which can prevent or mitigate cognitive deterioration [62]. Another behavior closely linked to habitual scrolling is "phubbing", which involves prioritizing smartphone use over interpersonal interactions, including within couples or families. The perceived stress caused by being "phubbed" can also diminish relationship satisfaction and, in turn, negatively affect sleep quality [63]. Implementing digital detox strategies may help relieve stress and improve well-being for both those who engage in phubbing and those who experience it, ultimately fostering stronger social connections and improving sleep quality. This effect would be especially pronounced when paired with relationship-focused and stress management interventions that directly address phubbing by partners, parents, or children [63].

Studies have reported that "digital detox" can positively affect a broad range of outcomes, such as reduced procrastination, boredom, stress, depression, and anxiety, as well as enhanced self-regulation, self-control, sleep quality, and overall life satisfaction, which were estimated soon after the intervention period, which often ranged from a week to a few months [65,68-70,73-75]. While some individuals may experience temporary feelings of alienation, craving, or increases in screen time for other activities (such as watching television), many adapt and ultimately report beneficial shifts in addictive behaviors and health-related measures [68,70,74]. Notably, unlike substance addictions, voluntary abstinence from smartphone and SM usage has been associated with only craving without an increase in negative mood or anxiety and rather an improvement in these negative affect states [70,74,75]. However, it is worth noting that excessive or inappropriate technology use, rather than typical, balanced usage, remains the primary risk factor for adverse outcomes, suggesting that targeted digital detox strategies can be effective tools for promoting mental health, preventing digital dementia, and supporting overall well-being [64,65,67,68,71].

Contextual factors that may modulate the impact of digital detox interventions: where do they matter the most?

The effectiveness and priority of digital detox interventions can vary widely depending on a range of contextual factors, including demographic characteristics (e.g., age and gender), baseline technology use, baseline well-being, and cultural context [62,65,70,71]. Adolescents and young adults, for example, undergo critical periods of brain development characterized by heightened neuroplasticity and susceptibility to immediate rewards, making them more vulnerable to negative outcomes from excessive screen time [62]. Moreover, initial findings in adolescent populations suggest that girls may derive greater benefit from digital detox interventions than boys [64], in line with literature showing heightened vulnerabilities and distinct developmental windows for depressive and anxiety symptoms as well as emotion regulation among female adolescents [76]. Conversely, targeted use of DT may offer cognitive benefits in older adults through specific applications. Additionally, working adults may benefit from more robust self-regulatory capacities but could face different challenges in balancing personal or professional demands [62].

In one of the most robust systematic reviews and meta-analyses to date on digital SM detox, Ramadhan et al. synthesized evidence from 12 studies in 2024, including randomized controlled trials (RCTs) and non-randomized experimental designs [65]. The RCTs were largely low-risk across key domains, and the non-randomized studies were moderate- to high-quality. This systematic review reveals both the potential benefits and the complexities of implementing digital detox interventions in diverse contexts [65]. While digital detox may significantly reduce depressive symptoms, the effect on broader outcomes such as stress, life satisfaction, and mental well-being is often inconclusive. This discrepancy appears partly attributable to heterogeneous intervention durations (e.g., short-term versus sustained reduction), definitional variations (e.g., total abstinence versus partial limits on specific platforms), and individual differences in baseline severity of symptoms, coping styles, and environmental pressures. For instance, individuals presenting with higher levels of depression at the outset tended to benefit more versus those with milder symptoms, where the effects were statistically and not clinically significant. Additionally, external stressors and local cultural factors (e.g., societal norms around technology use and lack of availability of offline social resources) could overshadow the positive impact of detox, highlighting the importance of tailoring interventions to specific demographic and psychological needs. These findings highlight the necessity for more targeted research, particularly subgroup analyses and longitudinal studies that can pinpoint which populations and settings stand to gain the most from digital detox strategies alongside the development of standardized measures that capture the complex nature of well-being beyond depressive symptomatology.

The geographical location (urban versus rural) and social environment (e.g., phubbing and relationship satisfaction) also appear to play a critical role in shaping individual responses to digital detox efforts. For instance, a narrative review from 78 high-quality studies noted that high-exposure urban settings may intensify digital dementia risk through attention deficits, memory loss, and academic challenges. In contrast, regions with less connectivity might see different usage patterns or priorities [62]. Meanwhile, as phubbing behaviors can negatively affect sleep quality by increasing perceived stress and lowering relationship satisfaction, digital detox strategies could facilitate emotional and social support [63]. Together, these studies suggest that cultural norms, socioeconomic factors, and the availability of emotional support and social connections can further influence whether a detox strategy will reduce stress and improve well-being or merely shift usage patterns to different platforms [62,64,67]. Moreover, current research often overlooks minority and marginalized populations, such as ethnic minorities and LGBTQ+ groups, or in rural settings for low-income nations, limiting the generalizability of existing findings [64,65,68].

Characteristics of effective digital detox interventions: how to implement?

Effective digital detox interventions share several key attributes, including appropriate duration, modality, and support mechanisms. For instance, some strategies emphasize short-term breaks (e.g., a few days of complete SM abstinence), whereas others advocate for more moderate but sustained reductions (e.g., limiting daily usage or disabling specific applications) [62,64,65,71]. Evidence suggests that personalized approaches, such as tailoring time limits based on individual habits and needs, can enhance adherence and reduce negative feelings (e.g., craving and loneliness) [66,69,70,72,73]. Incorporating support systems, whether through guided group sessions, professional counseling, or digital tools (e.g., monitoring apps such as ioS Screen Time, Forest, Android Digital Well-Being, Moment, Detox, Quality, Space, Pfftime, and notifications off), further boosts efficacy by helping users manage cravings and maintain motivation [69,73]. Additionally, pairing digital detox with alternative activities, such as mindfulness, exercise, or social engagement, improves emotional resilience and deter compensatory screen use [67,68]. Finally, interventions that explicitly address different life stages, parental controls for children, or relationship dynamics (e.g., partner phubbing) are more likely to yield sustainable changes in well-being [62,63]. Overall, a flexible, context-specific, and supportive approach, rather than a one-size-fits-all method, is crucial for successful digital detox outcomes [67].

Discussion on clinical implications and applications for digital detox based on findings of this review

Recent estimates suggest that children aged 8-12 spend about 4-6 hours each day on their devices, which increases to around nine hours per day for teenagers and young adults [77]. A growing body of evidence highlights the high comorbidity between PSU and common psychiatric disorders, especially with depression, but also with anxiety and obsessive-compulsive disorder (OCD) [10,78]. A meta-analysis of 17 case-control studies (18 datasets, 24,019 participants) found that 36.5% of university students had PSU, which was significantly associated with higher rates of depressive symptoms and suicidal ideation (odds ratio: 2.4 and 2.18, respectively) [79]. Another meta-analysis of 41 studies (41,871 participants) found that about one in four children and young people exhibit PSU, which was significantly associated with higher odds of depression, anxiety, perceived stress, and poor sleep quality (odds ratio: 3.17, 3.05, 1.86, and 2.60, respectively) [80]. The co-occurrence of PSU with numerous mental health disorders reinforces the importance of considering PSU in broader mental health assessment and treatment, as well as strategies for the prevention of PSU [36,78].

Regarding assessment, neither the International Classification of Diseases (ICD)-11 nor the Diagnostic and Statistical Manual of Mental Disorders (DSM)-5 officially recognize PSU or SM addiction as a standalone diagnosis, although they are identified as an area requiring further research [81-83]. It is possible that these problems will be considered in future guides for diagnostic formulations (e.g., gaming disorder is now included in the ICD-11). Lin et al. demonstrated that smartphone addiction shares the core DSM-5 diagnostic factors of substance and non-substance use disorders and have proposed the following diagnostic criteria for smartphone addiction: compulsive behavior, functional impairment, withdrawal, and tolerance [84]. However, although terms such as "regular use", "unhealthy use", "addiction", and "use disorder" are often used interchangeably, they refer to different concepts [77]. DSM-5 describes "use disorder" in the context of substance addictions based on criteria such as tolerance, cravings, withdrawal, and continued use despite negative consequences. SM use has risen significantly among children, adolescents, and young adults over the past decade, yet the study of behavioral addictions lags behind substance use disorder research [81].

In this developing field, the term "unhealthy use" has been described by Xu et al. for any behavior that raises the risk of harm, whether or not it meets the formal criteria for an addiction. Consequently, "unhealthy use" can serve as a practical term for identifying problematic screen behaviors and guiding preventive interventions [77], irrespective of diagnostic classifications. Should clinical criteria and DSM classification for PSU or problematic DT or SM use become established, "digital detox" would be a salient consideration in treatment protocols, following a similar trend to other interventions for behavioral addictions.

Treatment addressing PSU and SM addiction may need to consider a wide range of interrelated difficulties associated with technology use. Although there is a distinction between digital (a broader focus on all digital devices) and SM detox, the two concepts often overlap in literature [72]. For example, one of the studies included in this review among university students in Lebanon suggested that Instagram is among the most difficult platforms to quit, highlighting the distinct challenges posed by SM use relative to broader technology habits [72]. Hence, clinicians should approach youth openly and without judgment when discussing screen time and social media use, as disentangling unnecessary and potentially harmful use from the use required to engage in day-to-day experiences is incredibly complex. By cultivating a safe space to engage in simple conversations about the role of technology in a patient's life, for example, by asking if they have their own phone, how much time they spend online, how SM affects their mood, and if they experienced or witnessed cyberbullying, sexting, etc., clinicians may identify potential "unhealthy use' and determine whether a digital detox might be beneficial [77].

**Figure 2:**
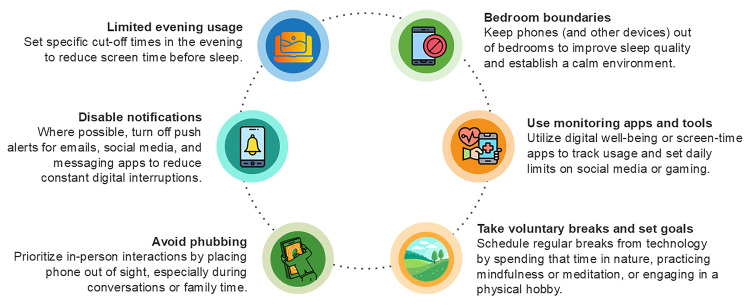
Digital detox strategies for everyday life Image credit: Dr. Sajita Setia (corresponding author)

Although digital detox remains a promising intervention, its efficacy may hinge on factors such as the severity of the user's dependence, supportive networks, and the individual's broader psychological context [85,86]. Collectively, findings from the studies reviewed here reinforce the importance of exploring diverse approaches to mindful use of DT and SM along with strategies to boost emotional health and social connections [36,64]. As shown in Figure 2, a variety of digital detox strategies can be integrated into treatment or used by individuals for everyday life.

In particular, pragmatic approaches to digital detoxes and aligned strategies, which meet individuals where they are, promise the most mental health benefits. Mindful digital downtime, which refers to strategically withdrawing from devices or apps, has been associated with reductions in depressive symptoms and other indicators of distress [65]. This approach could be most effective when individuals focus on limiting the most problematic apps (e.g., TikTok and Instagram) [72,85-86] along with graduated and strategic reductions in DT usage instead of uniform abstinence [67]. Education and counseling on both the potential harms of unhealthy use of DT and the benefits of digital detox can further empower families and youth to make informed decisions and adopt healthier technology habits [87]. This approach helps them develop self-regulation skills and gradually assume greater autonomy over technology use [87].

Integrating digital detox strategies into existing mental health services could also help people develop healthier coping mechanisms, particularly those with pre-existing depression, anxiety, attention deficits, or mild cognitive impairments linked to excessive screen use [62,64,65]. Mindfulness-based techniques in therapy sessions and support groups should address both the habit of overreliance on technology and the underlying emotional or cognitive vulnerabilities that reinforce it [67,68]. In other words, treatment should address the etiology of multiple co-occurring issues and integrate consideration of the ways in which they transact in case conceptualization.

Relationship counseling also offers a promising avenue for exploring digital detox as a way to counteract phubbing. Studies have demonstrated that partner, adolescent, or parental phubbing is linked to diminished relationship satisfaction, heightened stress, and poor mental health and sleep quality [63,88-91]. In clinical practice, it is essential to offer non-judgmental counseling and involve families in shared decision-making to promote healthier screen use [77]. When couples and families learn to set boundaries around technology, such as device-free mealtimes, limited evening usage, and removing all devices from bedrooms before bedtime, they may notice improvements in communication, emotional support, sleep quality, and well-being [87]. Incorporating digital detox strategies into relationship-focused therapy and workshops can address these relational strains and promote more meaningful interpersonal interactions [92-95].

Unmet needs for further research and policy implementation

**Figure 3:**
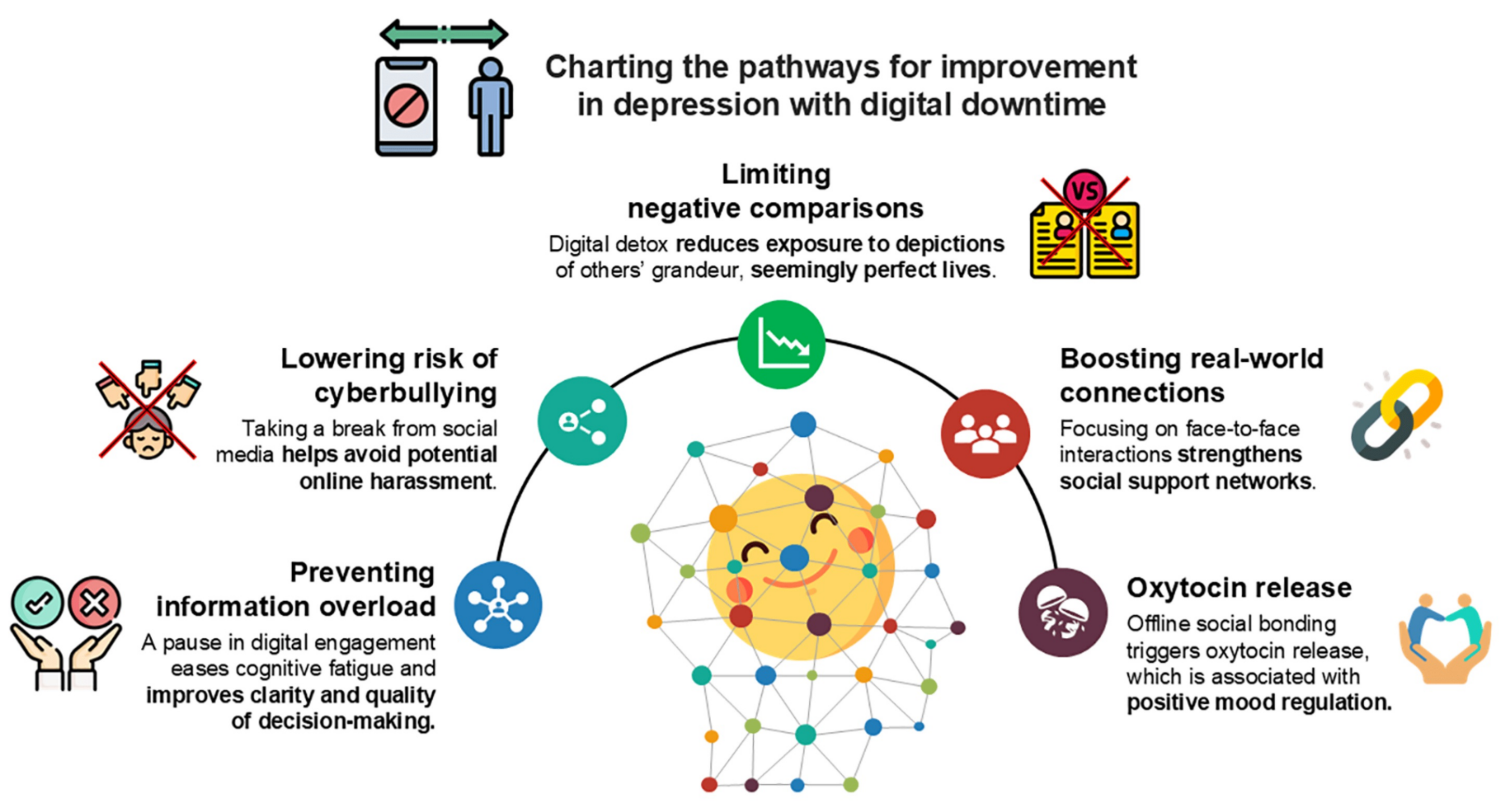
Charting the pathways: how does mindful digital downtime lead to a reduction in depression? Constant exposure to idealized online content can trigger social comparison, leading to increased cortisol levels and stress. Disconnecting from digital platforms may help lower cortisol levels, alleviating stress that contributes to depressive feelings [65]. Image credit: Dr. Sajita Setia (corresponding author)

In systematic reviews, scholars have examined the relationship between SM usage and life satisfaction and mental health outcomes, with some digital detox interventions reporting significant gains in mental well-being, while others observe no or minimal changes [38,65]. This discrepancy may arise from both a lack of high-quality research and an incomplete understanding of which populations benefit most. For example, a review by Radtke et al. predominantly included lower-quality studies [38], whereas Ramadhan et al. analyzed only high-quality research [65]. In the meta-analysis by Ramadhan et al., digital detox interventions significantly mitigated depression (95% CI: -0.51, -0.07; p = 0.01) despite varying intervention durations across different studies (ranging from a full week of SM abstinence to reducing daily usage by just 10 minutes over three weeks) [65]. Figure 3 illustrates the pathways proposed by Ramadhan et al., demonstrating how mindful digital downtime can reduce depressive symptoms.

Despite the growing body of work on digital detox as a potential remedy for issues such as digital dementia, psychological distress, and PSU, there remain substantial gaps in both research and practice [62,64,67,68,73]. Systematic and narrative reviews have noted that existing studies have small or non-representative samples (e.g., university students or participants from Western contexts only), with short intervention windows, and an absence of standardized tools for measuring outcomes such as stress, cognitive decline, or emotional well-being [62,64,65,71]. This heterogeneity in sample demographics, intervention duration, and assessment methods complicates direct comparisons across studies. Future research would, therefore, benefit from larger-scale, longitudinal, and more diverse studies, particularly those employing randomized controlled trials and studying the long-term sustenance of digital detox practices and outcomes. These studies should include populations with clinical-level digital dependence (e.g., those with severe withdrawal symptoms or comorbid mental health conditions) and marginalized groups.

Currently, there is no definitive professional guidance on daily screen time for children over the age of 12 years or on when older children should begin using phones or mobile devices with supervision [77,96]. Nonetheless, some policies across the globe exist. The Ministry of Health in Singapore issued updated guidelines on January 21, 2025, regarding screen use for children aged 0-12 years [96]. These guidelines emphasize more stringent recommendations for parents, including limiting screen time to <1 hour per day outside of school for children aged 3-6 years and to <2 hours per day for those aged 7-12 (except for school-related activities). The new guidance also advises parents against granting children unrestricted use of mobile devices or access to SM platforms. Research assessing the mechanisms employed to enact these policies, as well as the outcome of these policies, will be instrumental in understanding a path forward.

As more and more communities (e.g., schools, districts, states, and countries) seek to develop and enact policy, engaging and collaborating with key stakeholders in the process will be key. For instance, schools, universities, and workplaces could arrange critical thinking workshops to support media literacy, mindful use of technology, and emotional and social learning, ultimately encouraging persistent voluntary behavior changes. Public health initiatives can also focus on raising awareness of concepts such as digital dementia and phubbing to highlight the importance of balanced technology use across all age groups. However, the potential for community-grounded actions (e.g., commitment among parents and guardians to delay smartphone use in children and adolescents) must be coupled with research and health-oriented guidance so the onus is not left only to the public to address this critical area of concern.

Limitations

While evaluating the findings of our review, it is important to note that this is a comprehensive analysis using a focused keyword search within the PubMed database. This approach may have restricted the breadth of relevant studies captured. However, the decision to focus our search in this manner was strategic, given the comprehensive nature of this scoping review along with the relatively underdeveloped state of standardized terminologies in the field of digital detox [31] and the predominance of low-quality studies across other databases, as documented in a prior systematic review [29].

## Conclusions

Growing evidence has confirmed that PSU frequently co-occurs with common psychiatric conditions, including depression and anxiety. Hence, digital detox, a term often used and described in the literature as a targeted break or disconnection from technology, has gained growing attention as a potential strategy to improve mental health and overall well-being. Emerging evidence suggests that its clinical relevance for enhancing mental health is especially pronounced among adolescents, young adults, women, and individuals with pre-existing problematic internet use or mental health conditions. Education and counseling about the potential harms of unhealthy technology use and the benefits of digital detox can empower families and youth to make informed choices and cultivate healthier habits. It is also important to note that small sample sizes, short intervention periods, and limited follow-up constrain the implications of current literature on digital detox. Hence, further research is needed to explore how best to implement these strategies for scalable and sustained long-term benefits.
